# Evolutionary genomics of APSE: a tailed phage that lysogenically converts the bacterium *Hamiltonella defensa* into a heritable protective symbiont of aphids

**DOI:** 10.1186/s12985-021-01685-y

**Published:** 2021-11-10

**Authors:** Bret M. Boyd, Germain Chevignon, Vilas Patel, Kerry M. Oliver, Michael R. Strand

**Affiliations:** 1 Department of Entomology, University of Georgia Athens, Athens, GA USA; 2 Present Address: Center for Biological Data Science, Virginia Commonwealth University, Richmond, VA USA; 3 Laboratoire de Génétique et Pathologie des Mollusques Marins, IFREMER, La Tremblade, France

**Keywords:** Virus, Bacteria, Mutualism, Aphid, Parasitoid

## Abstract

**Background:**

Most phages infect free-living bacteria but a few have been identified that infect heritable symbionts of insects or other eukaryotes. Heritable symbionts are usually specialized and isolated from other bacteria with little known about the origins of associated phages. *Hamiltonella defensa* is a heritable bacterial symbiont of aphids that is usually infected by a tailed, double-stranded DNA phage named APSE.

**Methods:**

We conducted comparative genomic and phylogenetic studies to determine how APSE is related to other phages and prophages.

**Results:**

Each APSE genome was organized into four modules and two predicted functional units. Gene content and order were near-fully conserved in modules 1 and 2, which encode predicted DNA metabolism genes, and module 4, which encodes predicted virion assembly genes. Gene content of module 3, which contains predicted toxin, holin and lysozyme genes differed among haplotypes. Comparisons to other sequenced phages suggested APSE genomes are mosaics with modules 1 and 2 sharing similarities with *Bordetella-*Bcep-*Xylostella fastidiosa-*like podoviruses, module 4 sharing similarities with P22-like podoviruses, and module 3 sharing no similarities with known phages. Comparisons to other sequenced bacterial genomes identified APSE-like elements in other heritable insect symbionts (*Arsenophonus* spp.) and enteric bacteria in the family *Morganellaceae*.

**Conclusions:**

APSEs are most closely related to phage elements in the genus *Arsenophonus* and other bacteria in the *Morganellaceae*.

**Supplementary Information:**

The online version contains supplementary material available at 10.1186/s12985-021-01685-y.

## Background

Viruses that infect bacteria (phages) are the most numerous biological entities on Earth [1–3]. Phages also affect many important ecological and evolutionary processes through the mortality effects they have on bacteria [4, 5] and the horizontal transfer of genes that enhance bacterial fitness [6, 7]. Most known phages infect free-living bacteria that live in soil, water or other habitats but a few have been identified that infect heritable symbionts of eukaryotes [8–14].

Heritable symbionts associated with insects include obligate mutualists that provide essential benefits to their hosts, facultative mutualists that provide conditional benefits, and reproductive parasites [10, 15–30]. Aphids (Hemiptera:Sternorrhyncha:Aphidoidea) are insects that commonly harbor multiple heritable symbionts [10, 31–33]. Approximately one third of sampled aphid species also harbor the facultative mutualist *Hamiltonella defensa*, which is a γ-Proteobacterium (order Enterobacterales, family *Yersiniaceae*) that primarily lives extracellularly in the hemocoel. *H. defensa* also infects other sternorrhynchan hemipterans [34–41]. *H. defensa* conditionally enhances the fitness of the pea aphid, *Acyrthosiphon pisum,* and other species by conferring resistance to parasitoid wasps that kill hosts by laying eggs into their bodies [18, 40, 42–45].

Several strains of *H. defensa* have been identified that differ in the levels of parasitoid resistance they confer upon the pea aphid [46–49]. Resistance is associated with infection by a double-stranded (ds) DNA phage that was originally named *A. pisum* secondary endosymbiont (APSE) [8] and was later found to infect *H. defensa* in aphids and other hemipterans [12, 36, 38, 41, 44, 45, 50–54]. Multiple APSE haplotypes were identified that encode different toxin genes with potential roles in mediating resistance to parasitoids [38, 51, 55, 56]. In *A. pisum*, *H. defensa* strains infected by a haplotype named APSE3 confer high levels of resistance by killing *Aphidius ervi* wasps during the egg stage, while strains infected by APSE2 and APSE8 confer an intermediate level of resistance by killing wasps as eggs or larvae [40, 45, 57]. APSE1 is also associated with high resistance to *A. ervi*, but little is known about timing of wasp mortality [12]. *H. defensa* strains that are not APSE-infected confer no resistance to wasps while imposing fitness costs on aphids that suggest a shift from being a conditional mutualist to a parasite [57, 58].

APSEs that confer resistance to parasitoids are integrated into the main chromosome of *H. defensa* [40, 56, 59], which supports that lysogenic conversion [60] underlies the evolution of this bacterium into a protective symbiont of aphids. Persistence as a provirus also enables the vertical transmission of APSEs to aphid offspring when *H. defensa* cells are maternally acquired [46]. However, some APSEs replicate and produce virions that can horizontally transfer the viral genome to non-infected strains of *H. defensa* [8, 40, 58, 61], while some *H. defensa* strains have been horizontally transmitted between insect hosts by different mechanisms [31, 35, 50, 62–66].

Previous results support that an APSE phage infected the common ancestor of all known *H. defensa* strains [62, 67]. However, the evolutionary relationship of APSE to other phages are incompletely understood. Early results showed that APSEs produce short-tailed virions that morphologically resemble phages assigned to the family Podoviridae (order Caudovirales) while sequence analysis identified some genes with predicted functions in virion assembly that shared similarities with genes in the model podovirus *Salmonella enterica* P22 [8]. Subsequent studies further noted that virion assembly genes in P22 are organized into a syntenous module in several phages including APSEs, while the hosts for these phages were primarily restricted to γ-Proteobacteria in the order Enterobacterales [68, 69]. However, low amino acid identities for most virion assembly genes suggested APSEs are among the most divergent of these P22-like phages [69], while genome-wide nucleotide homology clustered APSEs separately due to dissimilarities outside of the virion-assembly module [70]. Isolation of *H. defensa* as a specialized symbiont of sternorrhynchan hemipterans has been posited as one factor contributing to APSE divergence [62, 71]. Previous findings [9, 72–75] together with more recent results [67] identified phage elements present in two other groups of insect symbionts, *Arsenophonus* (*Morganellaceae*) and *Sodalis* (*Pectobacteriaceae*), which suggest horizontal exchange events may also contribute to APSE divergence.

In this study, we identified APSEs (herein APSE1 MI47, APSE9 MI12, and APSE8 5D) in three additional strains of *H. defensa* (MI47, MI12, and 5D) from *A. pisum.* We then used these data with other previously sequenced APSE haplotypes to identify: 1) key features of APSE genomes that are shared with other tailed phages assigned to the Caudoviralis, and 2) discern potential evolutionary relationships outside of the phage elements that exist in *Arsenophonus* and *Sodalis* spp*.*

## Methods

### APSEs from *Hamiltonella defensa* used in this study

Complete genome assemblies for the A2C, AS3, NY26, ZA17, 5AT, MI47, MI12, and 5D strains of *H. defensa* were previously generated by establishing clonal *A. pisum* lines containing each strain and then establishing in vitro cultures of each strain from these aphid lines (Table 1) [30, 40, 56, 59], that allowed for single molecule real-time (SMRT) sequencing without amplification or contamination by DNA from the genomes of the aphid or another abundant endosymbiont (*Buchnera*) following previously described protocols for library preparation and sequencing [56]. Each *H. defensa* genome was then assembled as detailed by Chevignon et al. [56]. Each APSE genome in the above strains of *H. defensa,* the APSE1 genome that was sequenced by van der Wilk et al. [8], and the APSE MEAM and APSE MED genomes from the *H. defensa* MEAM and MED strains were aligned using MAFFT [76] in Geneious (v.10; Biomatters [77]). Protein similarities were assessed by extracting coding sequences (herein genes) followed by translation and alignment in MAFFT. Genes conserved across all haplotypes were named as designated by van der Wilk et al. [8] for APSE1 while genes present in some but not all haplotypes were named as designated in subsequent studies [8, 56]. To assess whether frame shifts observed in *p24* from APSE MEAM potentially disables virus production, we used genome sequencing depth as a proxy by accessing the whole-genome shotgun sequence library from the whitefly species in the *Bemisia tabaci* complex that is the host for *H. defensa* MEAM (SRR3180082) [38, 78] and converting the data from SRA format to fastq using the SRA-Toolkit v.2.4.2. We then estimated sequence coverage using bowtie2 v.2.2.6 (implementing the -end-to-end option) [79] by aligning and counting reads that mapped to APSE-MEAM or *H. defensa* with APSE manually removed (NZ_CP016303.1).

**Table 1:** APSE haplotypes with fully sequenced genomes examined in this study

Haplotype	Bacterial host	Insect host	Toxin	Data type	Assembly type	References	NCBI identifier
APSE1	*H. defensa*	*Acrythosiphon pisum*	*stxB*	Phage genome	Whole genome	[8]	NC_000935.1
APSE3 AS3	*H. defensa* AS3	*Acrythosiphon pisum*	*YD-repeat*	Contig/scaffold	Whole genome	[56]	NZ_CP017610.1
APSE2 5AT	*H. defensa* 5AT	*Acrythosiphon pisum*	*cdtB*	Contig/scaffold	Whole genome	[56]	NZ_CP001277.1
APSE2 NY26	*H. defensa* NY26	*Acrythosiphon pisum*	*cdtB*	Contig/scaffold	Whole genome	[56]	NZ_CP017605.1
APSE8 ZA17	*H. defensa* ZA17	*Acrythosiphon pisum*	*cdtB*	Contig/scaffold	Whole genome	[56]	NZ_CP17613.1
APSE MEAM	*H. defensa* MEAM	*Bemisia tabaci*	*cdtB*	Contig/scaffold	Whole genome	[38]	CCVN0000000
APSE MED	*H. defensa* MED	*Bemisia tabaci*	*cdtB*	Contig/scaffold	Whole genome	[51]	GCF_000258345.2
APSE1 MI47	*H. defensa* MI47	*Acrythosiphon pisum*	*cdtB*	Contig/scaffold	Whole genome	This study	NZ_CP022932.1
APSE9 MI12	*H. defensa* MI12	*Acrythosiphon pisum*	*cdtB*	Contig/scaffold	Whole genome	This study	CP023987.1
APSE8 5D	*H. defensa* 5D	*Acrythosiphon pisum*	*cdtB*	Contig/scaffold	Whole genome	This study	NZ_CP021663.1

### Candidate ortholog identification

We identified candidate orthologs by searching the NCBI non-redundant (nr) database using BLASTP [80] for each gene in the APSE3 AS3 prophage genome. Searches were limited to either entries classified as Caudovirales or γ-Proteobacteria. Results were downloaded as tab separated values and filtered to find the best hit for each gene predicted in the APSE genome. Additional searches were conducted to identify candidate homologs of genes absent in APSE3 AS3 genome, but present in other APSE genomes, including cytolethal distending toxin (*cdtB*) found in APSE8 ZA17 and shiga toxin (*stxB*) found in APSE1. Additional focused BLASTP and BLASTN searches of NCBI whole genome sequence (wgs) and RefSeq genomes databases were used to identify proviral elements that shared multiple homologs with APSE. BLAST searches were conducted during January of 2021.

### Identification of APSE-like elements in bacterial genomes

We used a TBLASTX search with APSE3 AS3 genes serving as queries to identify candidate APSE prophage elements in bacterial genomes. Here we searched NCBI RefSeq genomes and wgs databases targeting bacterial genomes classified as *Morganelleaceae*, *Enterobacteriaceae*, *Pectobacteriaceae*, *Yersiniaceae*, and *Erwiniaceae*. Once an APSE-like prophage element was discovered, we manually identified the ends of each by comparison with other APSE genomes and then ascertained the extent of shared homologous regions by visualizing alignments using BRIG [81]. We formally compared each phage-like element to APSE using whole genome TBLASTX (V.2.9.0+) and visualizing the results using EasyFig.py (V.2.2.3) [82]. We used CD-search to identify toxin-encoding genes in the newly described APSE genome from *Arsenophonus* species [83]. In one instance we found a partial APSE-like element within the genome assembly of *Arsenophonus nasoniae* str. DSM15247. To obtain a more complete assembly of this APSE-like genome we returned to the original *A. nasoniae* str. DSM15247 read library. We then used aTRAM to isolate and create a draft de novo assembly of the *A. nasonia*-APSE genome [84–86]. Multiple APSE genomes were used as baits for aTRAM to collect all possible variations. Contigs obtained from aTRAM with similarity to *H. defensa* APSE were assessed for similarity to the APSE3 AS3 genome. Next, we generated a reference-guided assembly of the *A. nasoniae*-APSE genome using Geneious and both the APSE3 AS3 and APSE8 ZA17 genomes from which we extracted a consensus assembly. We then used the recently re-sequenced *A. nasoniae* str. FIN genome (NCBI assembly GCF_004768525.1) that was generated using a combination of long and short sequence reads to further assess our reference guided assembly and to determine the relative position of the APSE-like elements in the *A. nasoniae* genome.

### Phylogenetic analyses

Orthologs of *p19*, *p24*, *p41,* and *p45* encoded by different APSEs plus other phages and phage elements in bacteria were identified using BLASTP with full-length sequences retained and short or partial BLAST hits being rejected. In addition to the fully sequenced APSE haplotypes that were the focus of this study, this analysis also identified orthologs in other APSE haplotypes that had previously been partially sequenced [55]. Candidate orthologs were downloaded as nucleotide sequences, checked for length, and pseudogenes were identified. We next aligned each set of orthologs including pseudogenes using Geneious V.10 progressive translation guided alignment (translation table 11, PAM250 match/mismatch scoring matrix, gap open penalty of 12, and gap extension penalty of 3). Alignments including pseudogenes were hand corrected to account for frame shifts that disrupted translation alignment processes. We then used RAxML to infer phylogenetic relationships, which uses the General Time Reversible (GTR) model of nucleotide substitutions with the option to model site heterogeneity using Γ and invariant sites [87]. We first used PartitionFinder2 to determine the best model among models available in RAxML and optimal partitions for estimating free parameters [88]. We used AIC_c_ to select the optimal substitution model when sample size is small (number of sites divided by maximum number of potential model parameters resulted in a low value; observed ranged from 36 to 120). The GTR + Γ models were found to be the best fit and optimal partitions were identified. We then implemented RAxML (HPC v.8.2.8; random seed = 12,345) to find the best tree with appropriate partition and model. Support for phylogenetic relationships was determined as the percent of 1000 bootstrap replicates that agreed with the best tree. Resulting trees were viewed and figures were generated using FigTree v.1.4.3 (http://tree.bio.ed.ac.uk/software/figtree/). We then repeated our phylogenetic analysis using RAxML and optimal model selection with pseudogenes removed. Since recombination could also impact our phylogenetic results, we conducted an additional phylogenetic analysis using the NeighborNet method in SplitsTree4 [89, 90] with alignments lacking pseudogenes.

## Results

### Genome organization and sites of integration are conserved among APSE haplotypes

**Fig. 1:**
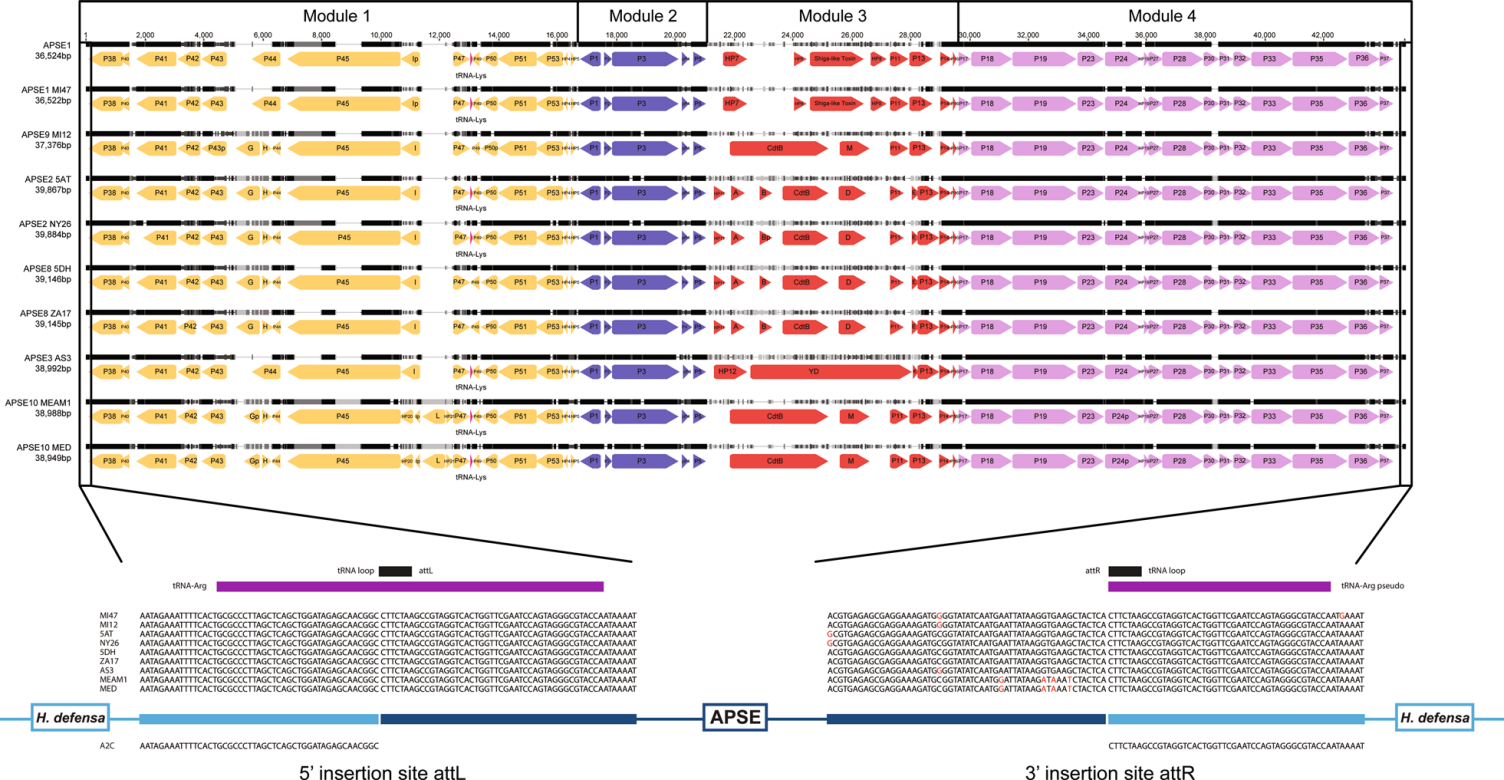
Genome alignment of sequenced APSE haplotypes. The upper part of the figure schematically shows the genome for each haplotype with total size in base pairs (bp) indicated to the left, the boundaries for each module indicated at the top and the boundary for the two predicted functional units indicated at the bottom. Arrows identify predicted genes and their orientation on the positive (right) or negative strand (left) and color (yellow, blue, red, lavender) indicating module assignment. Predicted functions for each gene are summarized in Table 2. The lower part of the figure illustrates the boundaries for the attL and attR sites for each haplotype. The purple bars indicate the left and right boundaries for the *H. defensa* tRNA-Arg gene, the black bars correspond to the position of the anticodon in the tRNA-loop, the light blue bars correspond to the *H. defensa* chromosome as identified by alignment to the *H. defensa* A2C strain, and the dark blue bars correspond to the left and right boundaries for the integrated APSE genome

Sequencing of the MI47, MI12 and 5D strains of *H. defensa* showed that each contained an APSE in the main host chromosome as a single copy provirus. We then used these APSE genomes together with other sequenced haplotypes [8, 38, 51, 56, 59] to compare overall features (Table 1). Total genome sizes ranged from 36,522 bp for APSE1 MI47 to 39,884 bp for APSE2 NY26 (Fig. 1). Predicted genes further ranged from a low of 41 for APSE3 AS3 to a high of 47 for APSE2 NY26, APSE8 ZA17, and APSE8 5D (Fig. 1). BLASTP searches against the NCBI nr database yielded predicted functions for 37 genes while 13 others were classified as unknowns or hypotheticals (Fig. 1, Table 2). Comparing each predicted gene across haplotypes indicated that APSE2 5AT and APSE2 NY26 were nearly identical to one another at the amino acid level (99.8%), whereas overall identities were lower when shared genes were compared to other haplotypes due to several genes including *p45, p36* and *p37* (Additional file 1: Fig. S1), which had been noted to vary among APSE haplotypes in other studies [67]. We also detected frameshifts in the major capsid protein gene (*p24*) in APSE MEAM and APSE MED that exist as proviruses in two *H. defensa* strains present in closely related whitefly species of the *Bemisia tabaci* complex named MED and MEAM [38]. These frameshifts suggested *p24* is pseudogenized in APSE MEAM and APSE MED which combined with previously identified defects in the regulator protein I and *p38* (integrase) from APSE MEAM and MED [38] suggest these prophages are inactive. To further investigate this possibility, we used publicly available shotgun sequencing data generated for *B. tabaci* MEAM to map reads corresponding to *H. defensa* and APSE. This analysis indicated *H. defensa* (340×) and APSE (431×) MEAM were sequenced to similar depth, which further supported this APSE haplotype persists as a prophage but likely produces no particles.

**Table 2:** Best hits identified to APSE3 AS3 coding sequences in other sequenced viruses or bacteria

Module	Locus	Alternative locus	Protein	Translation	Translation	BLASTP target: sequenced	Viral	Amino acid	BLASTP Target: sequenced γ-proteobacteria	Amino acid	BLASTP Target: all other	Amino acid
	Tag	Tag	Description	Start	Stop	viruses	family	Identity	That are insect symbionts	Identity	sequenced γ-proteobacteria	Identity
1	P38	APACPISMAS3_01	Integrase	1243	71	Proteus phage NV18	Podoviridae	76.68	*Arsenophonus* sp. ENCA	96.15	*Hafnia alvei*	73.78
1	P40	APACPISMAS3_02	Excisionase	1479	1099	Salmonella phage epsilon34	Podoviridae	44.72	*Sodalis glossinidius*	25	*Morganella morganii*	60
1	P41	APACPISMAS3_03	Dead-box helicase	2993	1611	Yersinia phage YeP4	Podoviridae	66.09	*Ca.* Arsenophonus triatominarum	93.9	*Providencia alcalifaciens*	81.4
1	P42	APACPISMAS3_04	DNA binding protein Roi	3744	3013	Cronobacter phage ENT47670	Siphoviridae	40.16	*Serratia symbiotica* str. Tucson	33.33	*Xenorhabdus* sp. PB62.4	54.58
1	P43	APACPISMAS3_05	Antirepressor	4591	3809	Salmonella phage SPN3UB	Siphoviridae	36.78	*Arsenophonus nasoniae*	87.31	*Salmonella enterica*	48.47
1	P44	APACPISMAS3_08	Nuclease	5166	4885	Xylella phage Xfas53	Podoviridae	43.01	*Arsenophonus nasoniae*	95.7	*Morganella morganii*	78.65
1	P45	APACPISMAS3_10	DNA polymerase I	7464	5401	Yersinia phage YeP4	Podoviridae	62.64	*Arsenophonus* sp. ENCA	92.87	*Vibrio vulnificus*	76.79
1	P46	APACPISMAS3_11	Transcriptional regulator	7809	7477	Escherichia phage D6	Myoviridae	30.53	*Arsenophonus nasoniae*	90	*Xenorhabdus* sp.	72.46
1	P47	APACPISMAS3_12	Phage protein	7900	8394	Xylella phage Xfas53	Podoviridae	37.3	*Ca.* Symbiopectobacerium PLON1	76.22	*Erwinia tracheiphila*	46.76
1	P49	APACPISMAS3_14	Phage protein	8685	8413	NA	NA	NA	*Arsenophonus nasoniae*	76.47	NA	NA
1	P50	APACPISMAS3_15	Phage protein	9308	8754	Yersinia phage YeP4	Podoviridae	59.02	*Arsenophonus* sp. Aphis craccivora	90.76	*Proteus mirabilis*	68.31
1	P51	APACPISMAS3_16	Phage protein	10,622	9324	Yersinia phage YeP4	Podoviridae	54.93	*Arsenophonus nasoniae*	89.58	*Vibrio vulnificus*	62.3
1	P53	APACPISMAS3_17	Phage protein	11,538	10,615	Yersinia phage YeP4	Podoviridae	34.05	*Arsenophonus* sp. Aleurodicus floccissimus	75.16	*Providencia alcalifaciens*	40.6
2	P1	APACPISMAS3_18	Repressor	12,804	12,106	Yersinia phage YeP4	Podoviridae	47.11	*Arsenophonus* sp. Aleurodicus floccissimus	89.66	*Citrobacter koseri*	50.22
2	P2	N/A	Transcriptional regulator	12,900	13,184	Yersinia phage YeP4	Podoviridae	57.89	*Arsenophonus* sp. Aleurodicus floccissimus	80.85	*Izhakiella capsodis*	42.05
2	P3	APACPISMAS3_20	ATPase	13,188	15,461	Yersinia phage YeP4	Podoviridae	67.89	*Arsenophonus* sp. Aleurodicus floccissimus	91.15	*Providencia alcalifaciens*	70.13
2	P5	APACPISMAS3_21	Antitermination protein Q	15,889	16,335	Stx2a-converting phage Stx2	Siphoviridae	61.67	*Arsenophonus* sp. Aphis craccivora	95.95	*Escherichia coli*	58.22
3	Toxin	APACPISMAS3_23*	YD-repeat protein	17,764	22,185	NA	NA	NA	*Arsenophonus* sp. Aleurodicus floccissimus	65.77	*Raoultella terrigena*	56.18
3	Toxin	APACPISM5AT_28*	*cdtB*	19,883	20,872	Enterobacteria phage cdtl	Siphoviridae	27.91	*Arsenophonus* sp. Bemesia tabaci	48.6	*Avibacterium paragallinarum*	29.91
3	Toxin	APACPISM1_23*	*stxB*	5398	6498	NA	NA	NA	NA	NA	*Photorhabdus* sp.	23.58
3	K	APACPISMAS3_24	Group II holin	22,837	23,028	Morganella phage IME1369_02	Siphoviridae	34.29	*Arsenophonus* sp. Bemesia tabaci	75.44	*Photorhabdus luminescens*	52.63
3	F	APACPISMAS3_25	Lysozyme	23,018	23,491	Salmonella virus BTP1	Podoviridae	52.15	*Arsenophonus* sp. Bemesia tabaci	85.99	*Serratia marcescnes*	56.34
3	P14	APACPISMAS3_26*	Exported protein	23,487	23,882	Enterobacteria phage SfV	Myoviridae	12.5	*Arsenophonus* sp. Bemesia tabaci	88.2	*Pantoea ananatis*	32.82
3	P16	N/A	Endolysin	23,911	24,030	Klebsiella phage ST16-OXA48phi5.4	Myoviridae	48.72	*Arsenophonus* sp. Bemesia tabaci	97.44	*Xenothabdus vietnamensis*	70.27
4	P17	APACPISMAS3_27*	Terminase, small	24,104	24,517	Providencia phage PSTNGR2lys	Siphoviridae	79.05	*Arsenophonus nasoniae*	89.71	*Providencia stuartii*	80.95
4	P18	APACPISMAS3_28	Terminase, large	24,534	25,934	Shigella virus Sf6	Podoviridae	38.7	*Ca.* Arsenophonus triatominarum	91.24	*Thiolinea disciformis*	54.2
4	P19	APACPISMAS3_29	Portal protein	25,940	28,105	Shigella virus Sf6	Podoviridae	70.9	*Ca.* Arsenophonus triatominarum	88.97	*Providencia alcalifaciens*	87.99
4	P23	APACPISMAS3_30	Scaffolding protein	28,156	29,052	Shigella virus Sf6	Podoviridae	58.94	*Arsenophonus nasoniae*	79.53	*Providencia rettgeri*	72.24
4	P24	APACPISMAS3_31	Major capsid protein	29,063	30,334	Salmonella virus HK620	Podoviridae	79.2	*Ca.* Arsenophonus triatominarum	93.6	*Providencia alcalifaciens*	85.07
4	P27	APACPISMAS3_33	DNA stabilization protein	30,554	31,036	Salmonella virus HK620	Podoviridae	58.13	*Ca.* Arsenophonus triatominarum	82.5	*Providencia rettgeri*	77.78
4	P28	APACPISMAS3_34	DNA stabilization protein	31,008	32,423	Salmonella phage SPN9CC	Podoviridae	60.81	*Arsenophonus nasoniae*	83.65	*Morganella* sp.	63.56
4	P30	APACPISMAS3_35	DNA stabilization protein	32,423	32,779	Proteus phage NV18	Podoviridae	38.79	*Arsenophonus* sp. Aleurodicus floccissimus	49.38	*Providencia alcalifaciens*	42.86
4	P31	APACPISMAS3_36	Hypothetical protein	32,779	33,246	Proteus phage NV18	Podoviridae	36.3	*Arsenophonus* sp. Aleurodicus floccissimus	84.52	*Proteus mirabilis*	35.95
4	P32	APACPISMAS3_37	DNA transfer protein	33,224	33,853	Escherichia phage vB EcoP Kapi1	Podoviridae	63.24	*Arsenophonus* sp. Aleurodicus floccissimus	82.16	*Escherichia coli*	63.55
4	P33	APACPISMAS3_38	DNA transfer protein	33,866	35,254	Salmonella virus BTP1	Podoviridae	33.13	*Arsenophonus* sp. Aleurodicus floccissimus	83.69	*Salmonella enterica*	65.34
4	P35	APACPISMAS3_39	DNA transfer protein	35,254	37,131	Salmonella virus P22	Podoviridae	64.03	*Arsenophonus* sp. Aleurodicus floccissimus	87.28	*Klebsiella pneumoniae*	68.43
4	P36	APACPISMAS3_40	Tail fiber protein	37,160	38,179	Proteus phage NV18	Podoviridae	72.27	*Arsenophonus nasoniae*	67.34	*Proteus mirabilis*	74.36
4	P37	APACPISMAS3_41*	Tail fiber assembly protein	38,158	38,625	Shigella phage SfIV	Myoviridae	32.37	*Arsenophonus nasoniae*	72.26	*Acinetobacter baumannii*	50.34

Each predicted coding sequence in the APSE3 AS3 genome, along with *stxB* and *cdtB* genes from APSE1 and APSE8 5AT, were used to search using BLASTp all submitted genomes for Caudoviruses and γ-proteobacteria that are either insect symbionts or not insect symbionts

*Denotes differences in predicted transcriptional start and stop exist between annotations of APSE

Other studies have noted that gene content and order are largely conserved among APSEs [36, 55, 67], but had not assessed genome organization from the perspective of functional units and module composition, which are characteristic of particular phage groups and suggest evolutionary constraints that maintain certain genes together because of their interactive roles in genome replication, lysogeny, virion formation and other essential functions [68–70, 91–100]. Examining our data set from these perspectives indicated that APSEs are organized into two functional units with early genes that have functions in integration, lysogeny and replication (module 1 plus *p1* in module 2) being on the negative strand and late genes with functions in genome packaging (*p2–p5* in module 2), virulence (module 3) and virion assembly (modules 4) being on the positive strand (Fig. 1, Table 1).

Integrases in temperate phages regulate site-specific recombination between the phage (*attP*) and bacterial (*attB*) attachment sites [101]. tRNA genes or sequences adjacent to tRNA genes are also common tailed-phage integration sites [102]. APSE phage (*attP*) and bacterial (*attB*) attachment sites were previously identified, with the latter occurring in a single copy tRNA-Arg gene [55]. By comparing each APSE-infected strain of *H. defensa* to the APSE-free A2C strain we showed that this site of integration (*attB*) was identical among examined strains (Fig. 1). Phage attachment *attP* core sequences were also almost identical among APSE haplotypes and located in a non-coding region between *p37* (domain 4) and *p38* (domain 1) (Fig. 1). Integration of each haplotype disrupted the host tRNA-Arg gene, but comparisons to the A2C genome showed that the left (*attL*) and right (*attR*) boundaries of the APSE genome complemented the *H. defensa* tRNA-Arg sequence, which repaired the host gene (Fig. 1).

Our naming scheme differed from Roüil et al. [67] who named APSEs on the basis of gene content in the toxin domain (module 3) and variation in a domain upstream of the DNA polymerase (*p45*) in module 1. This resulted in APSE8 being classified as a subtype of APSE2. In contrast, whole genome comparisons across all four modules underlies why we continued to distinguish APSE2 from ASPE8 as distinct haplotypes. For the same reasons, we called the phage variant from MI12 *H. defensa* strain APSE9 as it too was distinct from other named APSEs across modules.

### Modules 1, 2 and 4 share features with other phages

**Fig. 2:**
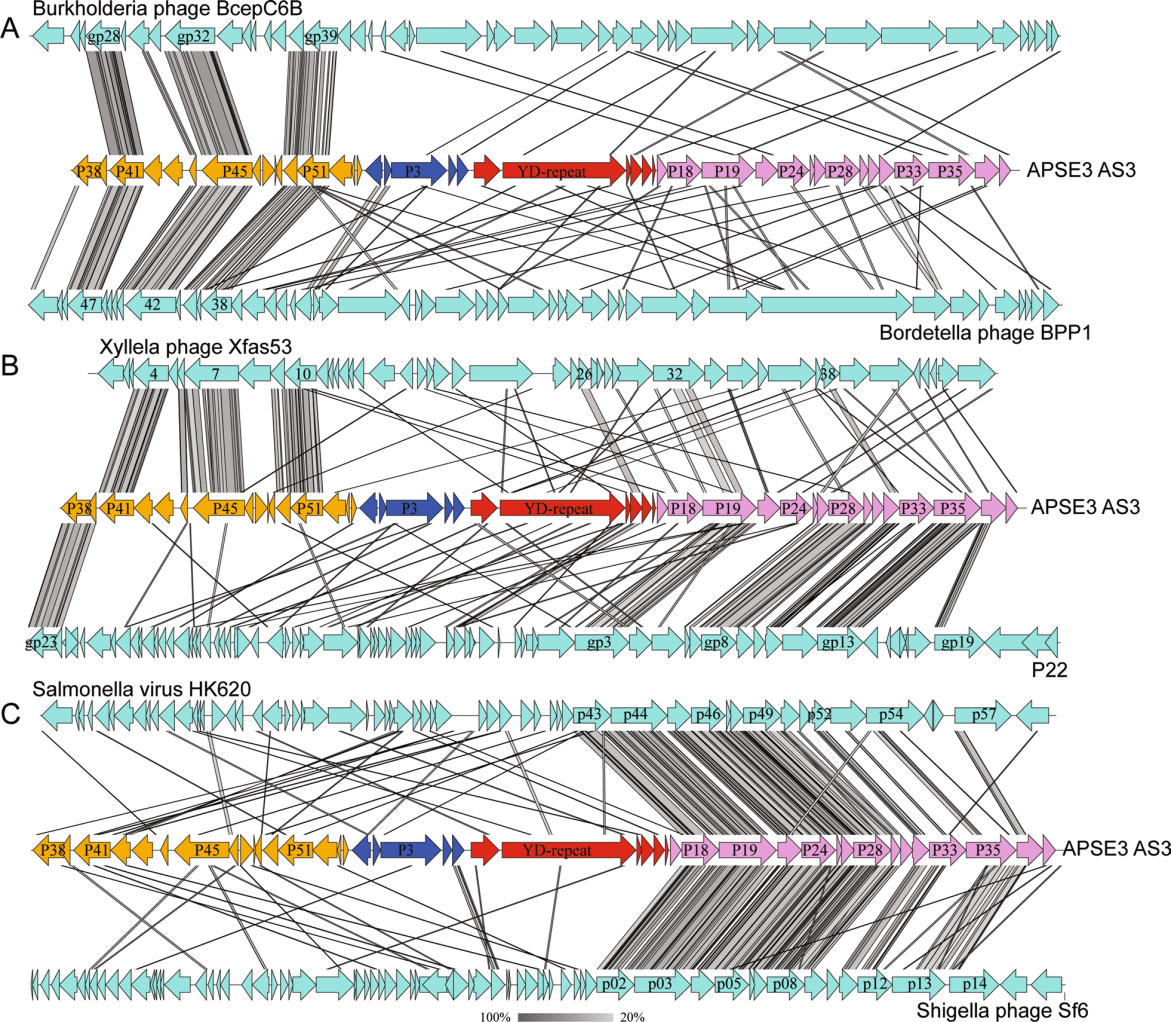
Comparison of the APSE3 AS3 genome to: **A** Salmonella virus HK620 and Shigella phage Sf6, **B** Xylella phage Xfas53 and Salmonella enterica phage P22, and **C** Burkholderia phage BcepC6B and Bordella phage BPP1. For APSE3, color-coded arrows indicate orientation of predicted coding sequences and module assignment as shown in Fig. 1, while coding sequences for the other phages are indicated by light blue arrows. Shaded bars connecting linear genomes define similar regions with the scale bar shown in the lower right of the figure defining TBLASTX identity

Given the conservation in gene order and content of modules 1, 2 and 4, we selected APSE3 AS3 as a model haplotype and used BLASTP to ask if any genes shared > 60% identity with predicted products from other fully sequenced viruses. Three genes in module 1, two genes in module 2, and seven genes in module 4 were identified that met this criterion, with each best hit being to another phage assigned to the families Podoviridae or Siphoviridae (Table 2). TBLASTX analysis corroborated previously noted similarities in gene order and content between the virion assembly module of APSEs (module 4) and P22-like phages that infect bacteria in the order Enterobacterales [68] including *Salmonella* virus HK620, *Shigella flexneri* phage Sf6, *Morganella* phage NV18, and *S. enterica* P22 [14, 71, 103–105] (Fig. 2A, B). Few similarities were detected between APSEs and these P22-like phages outside of their virion assembly modules (Fig. 2A, B), but similarities in gene order and content were identified between APSE module 1 and 2 in podoviruses that infect hosts outside of the Enterobacterales including *Xylella* phage Xfas53, *Burkholderia* phage complex members such as BcepC6B, *Bordetella* phage BPP-1, and *Yersinia enterocolitica* phage YeP4 [105–107] (Table 2; Fig. 2B, C). In contrast, no APSE genes in module 3 shared a similar level of sequence similarity with other known viruses.

### Other Enterobacterales besides *Arsenophonus* spp. contain APSE-like genes

We also assessed whether high identity homologs existed in any sequenced bacteria outside of *H. defensa* since this could suggest the presence of APSE-like prophages or prophage elements. We first considered other aphid symbionts, which like *H. defensa*, reside in the order Enterobacterales. These included several *Arsenophonus* spp. (*Morganellaceae*) and a *Sodalis* sp. (*Pectobacteriaceae*) that were already known to encode APSE-like genes [67, 72, 75]. We also included *Buchnera* and *Pantoea* (*Erwiniaceae*), *Regiella* and *Fukatsuia* that form a clade with *H. defensa* within the *Yersiniaceae* [30]*,* and *Serratia* that is also in the *Yersiniaceae.* Best hits (25–96% identities) using BLASTP were largely restricted to *Arsenophonus* spp., but included three genes from *Sodalis glossinidius*, *Ca*. Symbiopectobacterium, and *Serratia symbiotica* (Table 2). Extending this analysis to other Enterobacterales further identified high identity (> 60%) hits to APSE genes in four genera (*Xenorhabdus, Morganella, Proteus,* and *Providencia*) from the family *Morganellaceae.* Only four APSE genes (*p19*, *p23*, *p27*, *p28*) shared > 60% identity outside of γ-Proteobacteria with best hits to each being to *Mycobacterium tuberculosis* (Actinomycetales).

**Fig. 3:**
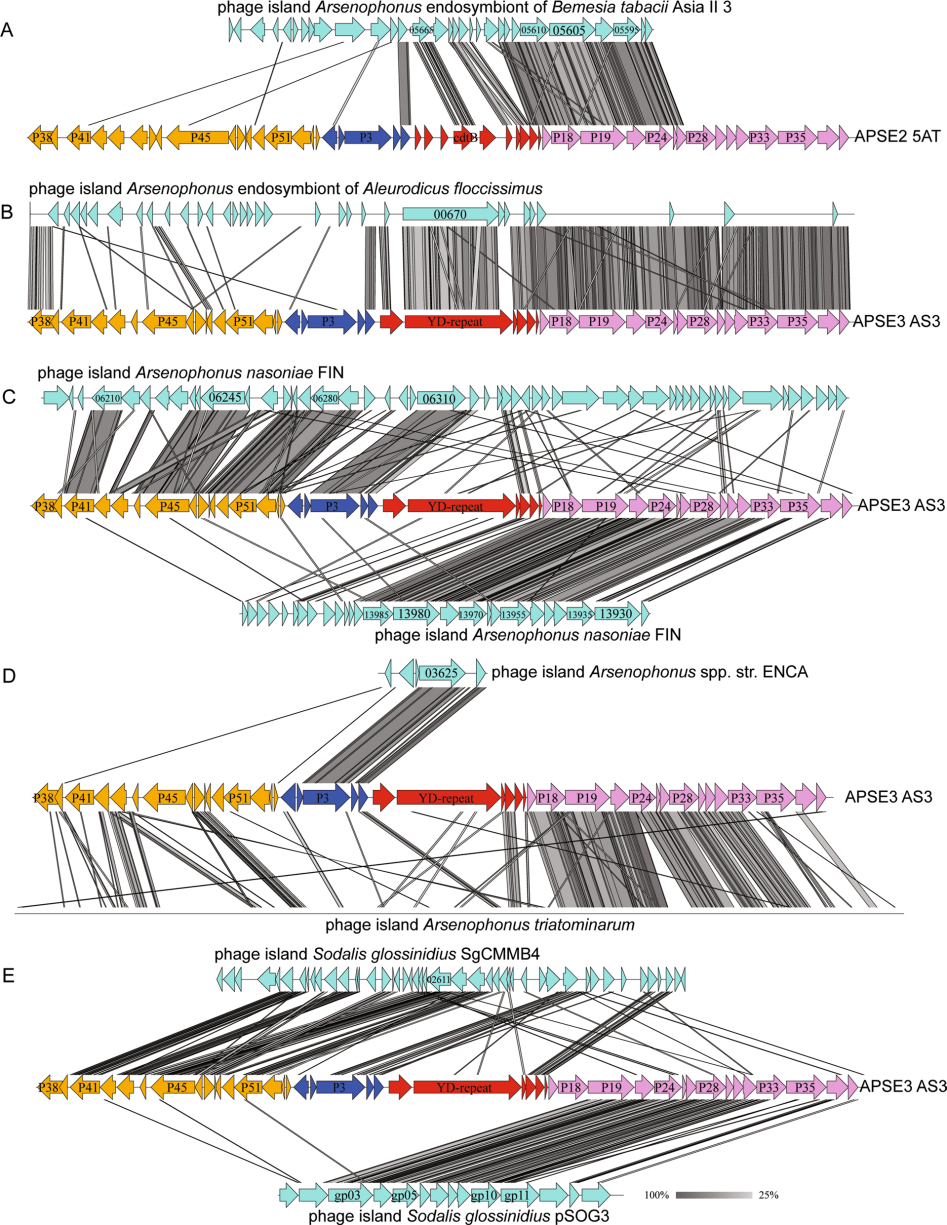
Comparison of the APSE2 5AT or APSE3 AS3 genome to phage elements present in the genomes of: **A** *Arsenophonus* spp. endosymbiont in of *Bemesia tabacii*, **B** *Arsenophonus* endosymbiont in the whitefly *Aleurodicus floccissimus*, **C** *Arsenophonus nasoniae* present in the wasp *Nasonia vitripennis,* **D** *Arsenophonus* spp. ENCA and *Arsenophonus triatominarum*, and **E** *Sodalis glossinidius.* Predicted coding sequences and shaded bars connecting APSE genomes to identified phage elements are defined as described in Fig. 2. Annotations were not available for *Arsenophonus triatominarum*

Given the preceding results, we interrogated the de novo genome assemblies available for four of the *Arsenophonus* spp., in which high identity APSE homologs were detected, from the perspective of both gene content and synteny. A single, small contig in *Arsenophonus* sp. str. ENCA contained a small syntenic region with coding sequences whose translation products shared high identities with products of the *p3, p4* and *p5* genes in APSE module 2 (Additional file 1: Table S1; Fig. 3A). In *Arsenophonus* sp. ex. *Aleurodicus floccissimus,* one contig contained a colinear block consisting of *p5, yd repeat* and homologs of all genes in conserved order for APSE module 4 (*p17–p37*), while a single contig was identified in *Arsenophonus* sp. ex. *Bemisia tabaci* Asia II 3 that contained *p5, cdtB,* and most genes (*p17–p28*) in conserved order for APSE module 4 (Additional file 1: Table S1; Fig. 3B). We recognized that the assemblies for these *Arsenophonus* spp. could have captured only part of an APSE genome given each derives from short read data and cannot be fully assembled. We therefore asked if reanalysis could generate additional information*.* We could only access original data for *A. nasoniae* DSM15247 which consists of short reads generated by Wilkes et al. [74] plus recently generated long read data from *A. nasoniae* FIN (NCBI SRA SRS441142 and SRX301737). The new assembly we generated using these data, with APSE3 AS3 and APSE8 ZA17 as references, unambiguously identified two syntenic domains. The first consisted of most but not all genes in APSE modules 1 and 2 in conserved order plus *f* (lysozyme) and *p14* in module 3, while the second domain contained *p14* plus most genes in module 4 (*p17–p33*) that were also in near fully conserved order (Fig. 3C). However, more than 1 Mb of *A. nasoniae* DNA was present between these domains and the prophage element with extensive similarity to APSE modules 1 and 2 was associated with virion assembly genes not found in APSE. Reexamining the plasmid pSOG3 from *S. glossinidius* str. *morsitans* showed that six virion assembly genes (module 4) shared > 60% amino acid identities with corresponding APSE3 genes in module 4 (Additional file 1: Table S1; Fig. 3D). However, all other genes on this plasmid were unrelated to APSEs. The *S. glossinidius* str. *morsitans* main chromosome contained a second domain encoding genes that shared significant identities with predicted APSE proteins in modules 1–3 but gene order only weakly resembled an APSE due to the presence of several unrelated bacterial genes or viral genes from other phages (Additional file 1: Table S1; Fig. 3D).

**Fig. 4:**
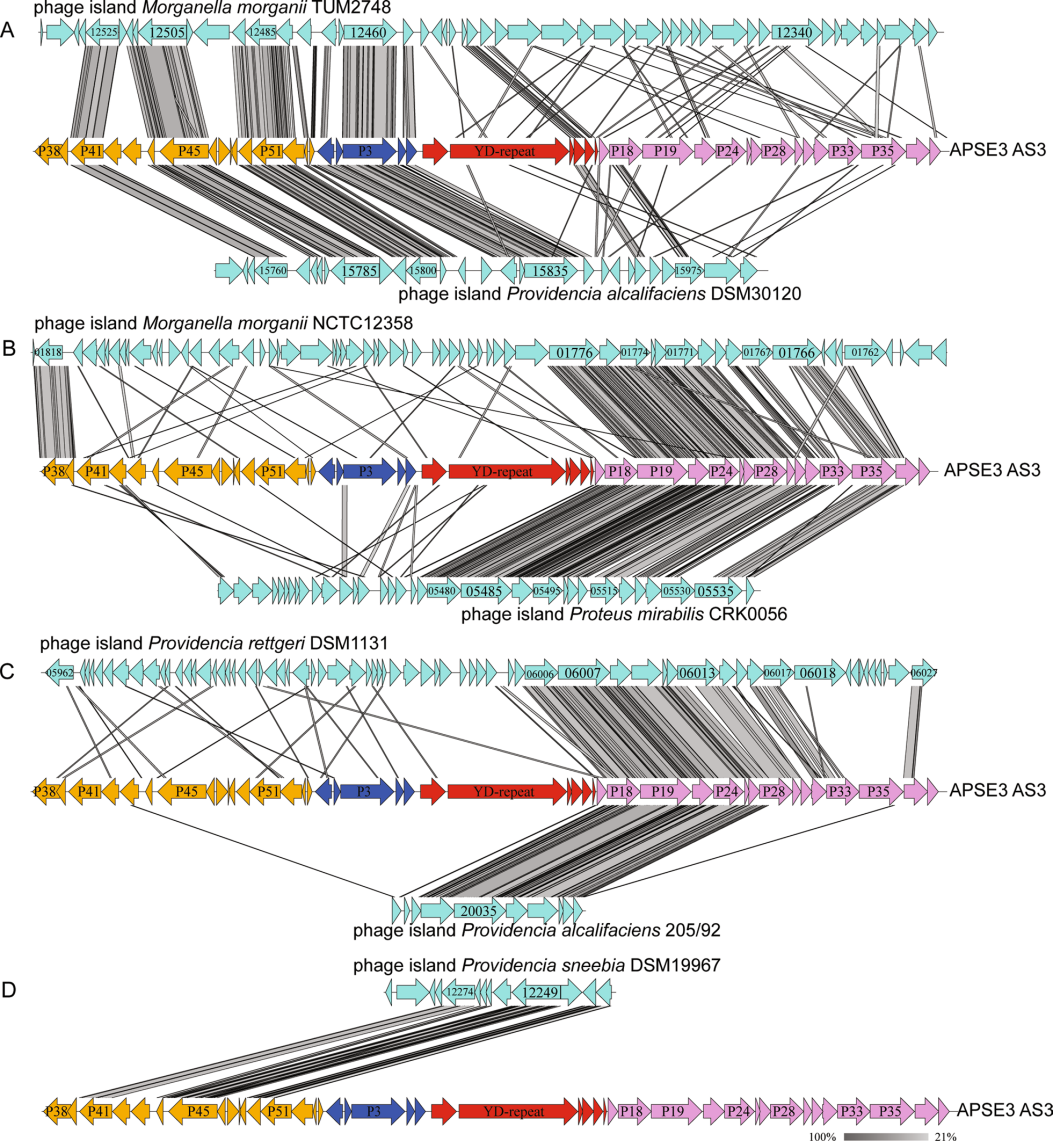
Comparison of the APSE3 AS3 genome to phage elements present in the genomes of: **A** *Morganella morganii* and *Providencia alcalifaciens*, **B** *Morganella morganii *and *Proteus mirabilis*, **C** *Providencia rettgeri*, and *Providencia alcalifaciens,* and **D** *Providencia sneebia*. Predicted coding sequences and shaded bars connecting APSE genomes to identified phage elements are defined as described in Fig. 2

Similar analysis of non-symbiont bacteria in the *Morganellaceae* detected homologs of APSE genes in colinear blocks corresponding to APSE module 1 and 2 plus a partial module 3 containing holin-lysozyme genes in the genomes of *Morganella morganii* (this region was associated with virion assembly genes not found in APSE) and two *Providencia* species (Additional file 1: Table S1; Fig. 4A). Colinear blocks corresponding to APSE module 4 were also identified in *M. morganii*, *Proteus mirabilis*, and two other *Providencia* species (Additional file 1: Table S1; Fig. 4B). In contrast, no colinear blocks or recognizable homologs were identified in these species that corresponded to APSE module 3 outside of *p14* and *p16*. Close inspection of the three intact phages (MmP1, MP1, and MP2) that have been identified from *M. morganii* [108, 109], four intact phages (PM16, PM75, PM87, and PM93) that have been identified from *Proteus mirabilis* [110] and a single phage (PR1) identified from *Providencia rettgeri* [111] indicated that none shared genes or sequence homology with APSEs. A BLASTN search failed to find any additional phages that have been deposited into NCBI which contain APSE-like modules like those present in *M. morganii*.

Altogether, no fully intact APSE-like genomes were identified outside of *H. defensa*, but colinear blocks containing high identity genes in syntenic order that corresponded to APSE modules 1, 2 and 4 were in both *Arsenophonus* spp. that are insect symbionts and certain other species in the *Morganellaceae* that were not. However, the only bacterium outside of *H. defensa* that contained a largely intact APSE-like toxin domain (module 3) was *Arsenophonus* sp. ex. *Aleurodicus floccissimus*.

### Phylogenetic analyses

**Fig. 5:**
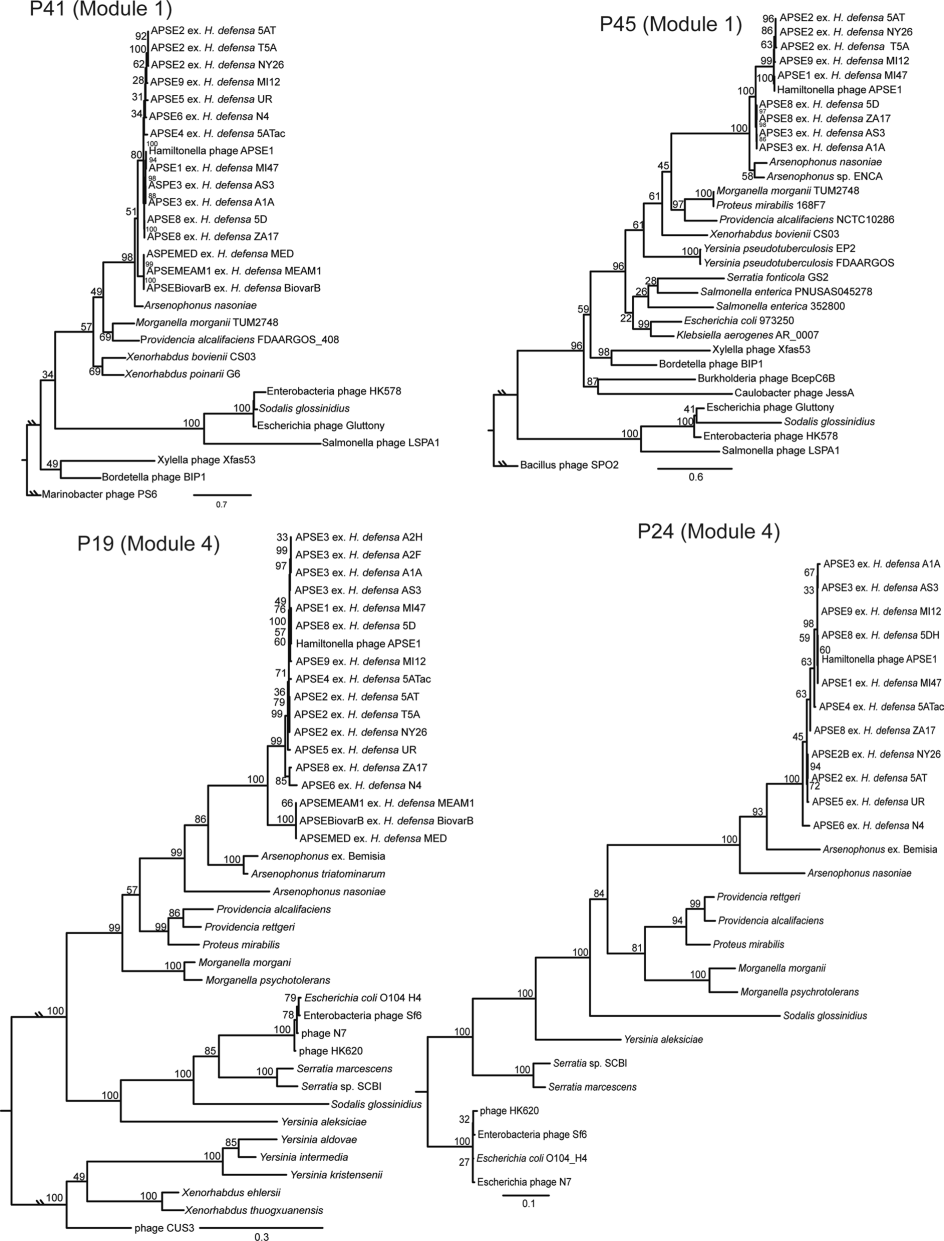
Maximum-likelihood phylograms depicting evolutionary relationships for two genes in module 1 (*p41* and *p45*) and two genes in module 4 (*p19* and *p24*) of APSEs, other phages and other phage elements from bacteria in the order Enterobacteriales. Numbers at nodes indicate percent of 1000 bootstrap replicates that recovered the same node. For each gene, tip labels indicate the APSE haplotype, sequenced phage, or bacterium containing a prophage element in which the ortholog resided. Scale bars indicates nucleotide substitutions per site. Tick marks indicate branches lengths at the root have been reduced

Since phage elements with similar gene order in APSE modules 1 and 4 were identified in some symbiont and enteric species of Enterobacterales, we generated phylogenies using two module 1 genes, *p41* (helicase) and *p45* (DNApol), and two module 4 genes, *p19* (Portal protein) and *p24* (Major capsid protein). These genes were selected to capture phylogenetic signal from each module and represented genes for which we could easily obtain orthologs from other phage and bacterial genomes, hence providing phylogenetic signal. APSE MEAM and APSE MED exhibit structural mutations in *p45* that suggest they are pseudogenized [38] while BLASTP identified frame shift mutations in *p19* from *Arsenophonus* sp. ex. *Aleurodicus floccissimus*. Phylogenetic trees further suggested inclusion of *p45* and *p19* pseudogenes generated phylogenetic error; namely long-branch attraction due to increased rates of nucleotide substitution in pseudogenes. We therefore conducted a phylogenetic analysis with pseudogenes removed. This analysis yielded several well-supported relationships (bootstrap support greater than 75%). Using genes from module 1 indicated all APSE haplotypes from *H. defensa* form a clade that is sister to APSE-like elements in *Arsenophonus* spp., while genes from module 4 found a similar pattern with homologs from APSE and *Arsenophonus* spp. being sister to prophage elements in *Morganella, Proteus*, and *Providencia* spp. (Fig. 5). Given the possibility for recombination events generating false phylogenetic signals [112, 113], we constructed phylogenetic networks using the same genes, which also supported that APSEs from *H. defensa* were closest to *Arsenophonus* spp. (Additional file 1: Fig. S2).

As previously noted, gene content in module 3 differs among haplotypes with APSE1, 4 and 5 encoding *stxB* toxin subunit genes, APSE2 5AT, APSE2 NY26, APSE6, APSE7, APSE8 ZA17, APSE8 5D APSE MEAM, and APSE MED encoding *cdtB* toxin subunit genes, and APSE3 AS3 encoding a *yd repeat* toxin gene [38, 51, 55]. We asked if *cdtB* represents a plesiomorphy within APSE phages. If *cdtB* diverged with APSE strains, we would expect the sequences in APSE2 5AT, APSE2 NY26 and APSE8 ZA17, APSE8 5D to share a similar number of identical DNA bases as APSE MEAM and APSE MED when compared to *cdtB* in, for example, *Arsenophonus* spp. ex *Bemisa tabaci* (in this case *cdtB* is a true homolog). However, if a *cdtB* moved by horizontal transfer from APSEs that infect *H. defensa* to the APSE-like phage element in *Arsenophonus* spp. ex *Bemisa tabaci* (or vice versa), then we would expect one of the *cdtB* genes in APSEs from *H. defensa* to be more similar to the *Arsenophonus* based *cdtB* gene (a paralog). We would further predict that *cdtB* in APSE MEAM/MED would also be more similar to the APSE-like *cdtB* gene in *Arsenophonus* spp. ex *Bemisa tabaci* given each infects the same whitefly host as *H. defensa* strains MEAM and MED. Results indicated that the percentage of shared identical nucleotides between *cdtB* in the phage element in *Arsenophonus* spp. ex. *Bemisia tabaci* Asia II 3 and APSEs were similar for both APSE MEAM/MED (46.1%) and APSE2/8 (47.7%), which was consistent with the *cdtB* genes representing a plesiomorphy in APSE (fig. S3).

## Discussion

The first studies of APSE genomes emphasized the variable content of virulence genes and their potential importance in converting *H. defensa* into a selective parasitoid pathogen [36, 55, 59, 62]. More recently, comparative data have identified other variable regions in APSE genomes while also showing that certain species of *Arsenophonus* contain APSE-like genes [67]. Results presented in this study further contribute to the APSE literature by showing that multiple haplotypes are organized into two functional units and four modules, with module order and gene order within modules 1, 2 and 4 being conserved. Our results also indicate that APSEs integrate into a conserved domain of the *H. defensa* genome while certain other phages and phage elements contain blocks of genes that share syntenic order with APSE modules 1, 2 and 4. While gene order and content of module 3 has previously been shown to differ among haplotypes [55, 67], our full genome comparison indicates the location of module 3 is located immediately downstream of the anti-termination protein Q gene (*p5*). This location is likely important, because virulence gene-containing modules in several other tailed phages that lysogenically convert host bacteria into pathogens are located in the same position [60, 113]. Genomic data for three *H. defensa* containing proviral APSEs that infect aphids in the genera *Cinara*, *Drepanosiphum*, and *Eriosoma* were also recently generated by short read sequencing [67]. We did not include these proviral APSEs in our formal analysis but inspection of these genomes indicates that gene order within modules 1, 2 and 4 are fully consistent with the APSE haplotypes that we analyzed.

Our results indicate that gene content and order of APSE module 4 is very similar to the virion assemble module of P22-like podoviruses [68, 70] while module 1 plus *p1* and *p2* (module 2) share syntenic order and identity with non-P22 like podoviruses that infect *Bordetella* spp. (BPP-1, BIP-1, BMP-1)*, Burkholderia* spp. (Bcep complex), *Xylella fastidiosa*, and *Yersinia enterocolitica* phage YeP4 [106, 107]. Thus, APSEs have mosaic genomes that consist of early *Bordetella-*Bcep-*X. fastidiosa-*like genes (module 1, 2) and late P22-like genes (module 4) with a centrally located toxin-holin-lysozyme domain (module 3) that shares no significant identity with other fully sequenced phages. APSE-like phages thus could have arisen through either module exchange between phages that infect disparate hosts or from a related phage with a similarly organized genome that has not been identified. The APSE-like domains in the genomes of *Arsenophonus* spp. were previously reported to not be intact [67], the results presented in this study indicate they contain syntenic regions that correspond to all of the APSE modules. That syntenic domains exist in several other species in the *Morganellaceae* further suggest APSE-like phages may infect bacteria that are not insect symbionts.

Our results support a close relationship between APSE and prophage elements in *Arsenophonus* spp., but cannot answer whether they represent related but independently acquired viruses or are evidence that host shifts have occurred between *H. defensa* and *Arsenophonus* symbionts. If we are correct that the *ctdB* genes in APSEs and APSE-like elements are orthologs, it is possible that the *ctdB* gene represents an ancestral state, that *yd repeat* and *stxB* genes represent replacements of the *cdtB* gene and that exchange of APSE or APSE-like phage between *H. defensa* and *Arsenophonus* has occurred since the acquisition of novel toxin genes. It is also possible that ancestral APSE-like phages contained different toxin genes, prior to the existence of modern *H. defensa*, and that multiple acquisition events have occurred, moving multiple toxin genes into *H. defensa.* Alternatively, our preferential detection of APSE-like prophage elements in *Arsenophonus* and certain other genera could reflect biases in the species of bacteria that have been sequenced to date. That APSE-like phages may infect bacteria in the Enterobacterales more broadly is supported by the detection of APSE-like elements during this study and previously [9] in *S. glossinidius str. morsitans* (*Pectobacteriaceae*) although weak synteny in gene order and overall low gene identities indicate severe decay of the ancestral APSE-like genome in this host species.

A limitation of using phylogenetic methods in discerning relationships of phage, is that recombination can obscure phylogenetic signal or create false relationships. One way in which this may be evident is disagreement between gene trees (*i.e.* gene tree-species tree conflict). In this study we selected two genes from the two larger modules, which had affinities to certain phage groups. This allowed us conduct phylogenetic analysis using identifiable orthologs shared between APSE and other genomes; however, we recognize that other gene trees could support an alternative arrangement, particularly when comparing between modules.

## Conclusions

APSEs have mosaic genomes that are organized into two functional units and four modules. Module order is conserved among haplotypes and the position of module 3, which encodes virulence factors, is likely important in converting *H. defensa* into a protective symbiont. APSE modules 1, 2 and 4 encode regulatory and structural genes, and these modules share syntenic domains with other phages and phage elements associated with symbiotic and non-symbiotic bacteria. We conclude that APSE arose through module exchange among phages, presently characterized or not, with similarly organized genomes.

## Supplementary Information

**Additional file 1**. Supplementary figures and table.

## Data Availability

Nucleotide alignments, phylogenetic trees, and prophage assembly from *Arsenophonus nasoniae* have been deposited in figshare https://doi.org/10.6084/m9.figshare.14744397. Genome assemblies of *Hamiltonella defensa* including proviral elements were obtained from NCBI https://www.ncbi.nlm.nih.gov/ and include CP017605-CP017610, CP022932-CP022937, CP021663-CP021665, and CP023987-CP023990. Raw sequencing data used in the assemblies of APSE genomes are available on NCBI and include SRR15674828-SRR15674831, SRR15676030, SRR1585572, and SRR15691200.

## Abbreviations

AIC: Akaike information criterion
APSE: *Acyrthosiphon pisum* Secondary endosymbiont
BLAST: Basic local alignment search tool
bp: Base pairs
*cdtB*: Cytolethal distending toxin encoding gene
DNA: Deoxyribonucleic acid
DNApol: DNA polymerase
ds: Double stranded
GTR: General time reversable model of nucleotide substitution
NCBI: National Center for Biotechnology Information
nr: Non-redundant
*stxB*: Shiga toxin encoding gene
tRNA: Transfer ribonucleic acid
Arg: Arginine
