# Supplementary material for: Evolutionary genomics of APSE: a tailed phage that lysogenically converts the bacterium Hamiltonella defensa into a heritable protective symbiont of aphids
Source: Virol J. 2021 Nov 10;18:219. doi: 10.1186/s12985-021-01685-y (PMC8579659; doi:10.1186/s12985-021-01685-y)
Supplement: Supplementary file 1 — Additional file 1. Supplementary figures and table. [file 12985_2021_1685_MOESM1_ESM.pdf]

Supplementary Tables and Figures

Evolutionary genomics of APSE: a tailed phage that lysogenically converts the bacterium *Hamiltonella defensa* into a heritable protective symbiont of aphids

Bret M. Boyd[1,2*], Germain Chevignon[3], Vilas Patel[1], Kerry M. Oliver[1], Michael R. Strand[1+]

[1]Department of Entomology, University of Georgia Athens, Athens, Georgia, USA

[2]Current address: Center for Biological Data Science, Virginia Commonwealth University, Richmond, Virginia, USA

[3]Laboratoire de Génétique et Pathologie des Mollusques Marins, IFREMER, La Tremblade, France

Correspondence: [+]mrstrand@uga.edu, [*]boydbm@vcu.edu

Table S1. Regions of the bacterial genomes that share homology with APSE genomes.

Target species	Identification	Genomic region, contig, or chromosome
Arsenophonus sp. str. ENCA	NHNG01000131	scaffold 83; 5,323 bp
Arsenophonus sp. ex. *Aleurodicus floccissimus*	OUND01000001.1	loci WBAF_RS00520-WBAF_RS00780
Arsenophonus sp. ex. *Bemisia tabaci* Asia II 3	NZ_MASH01000127	scaffold 273; 20,407 bp
Sodalis glossinidius str. *morsitans*	DQ785801.1	pSOG3
Sodalis glossinidius str. *morsitans*	LN854557.1	SgGMMB4
Morganella morganii	TUM2748	loci DDA57-RS12540-DDA57-RS12300
Morganella morganii	NCTC12358	loci NCTC12358_01818-NCTC12358_01758
Providencia alcalifaciens	DSM30120	loci PROVALCAL_03397-PROVALCAL_03428
Providencia alcalifaciens	205/92	HMPREF1563_RS20015-HMPREF1563_RS20055
Providencia sneebia	DSM19967	loci OO7_12289-OO7_12234
Proteus mirabilis	CRK0056	loci B9475_005380-B9475_005540
Providencia rettgeri	DSM1131	loci PROVERT_06006-PROVERT_06027

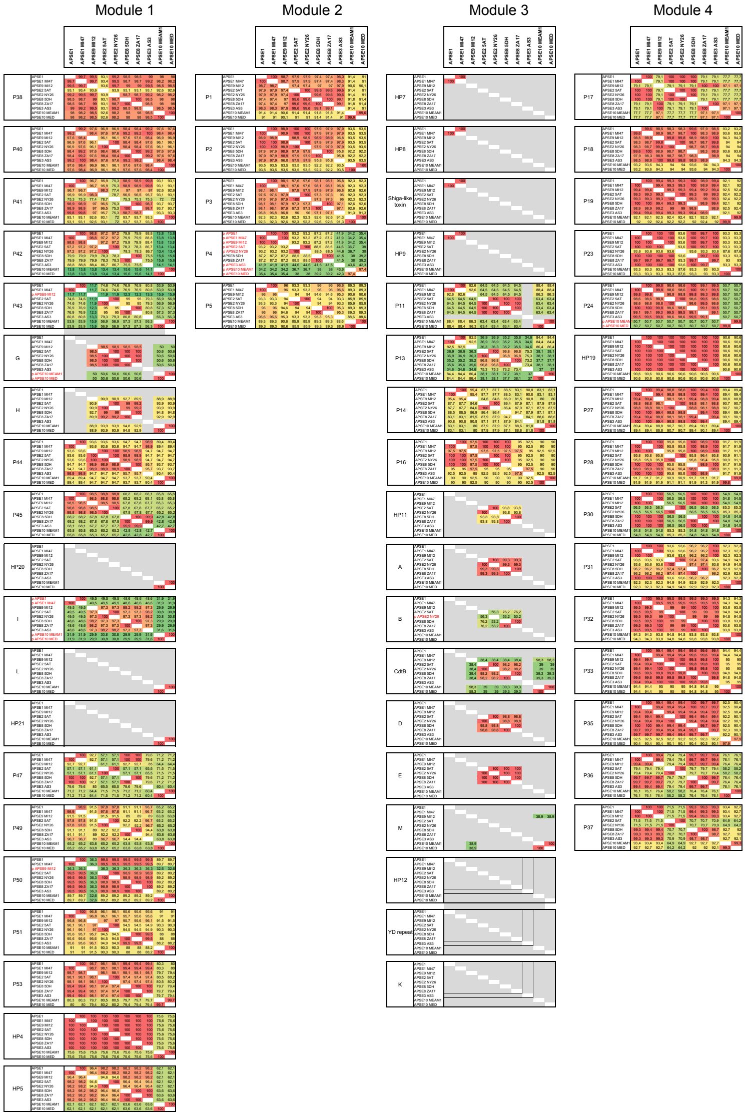

Figure S1. Protein identity matrix for predicted genes in modules 1-4 for each sequenced APSE haplotype. Matrix identity tables were made for each predicted protein from amino acid alignments. Identities were color coded from low (green) to high identity (red). Note that predicted gene products in module 1 (G, H, HP20, L, HP21) and most predicted gene products in module 3 are present in only some haplotypes or a single haplotype (see Fig. 1 and main text) with grey backgrounds indicating that no identity comparison could be made.

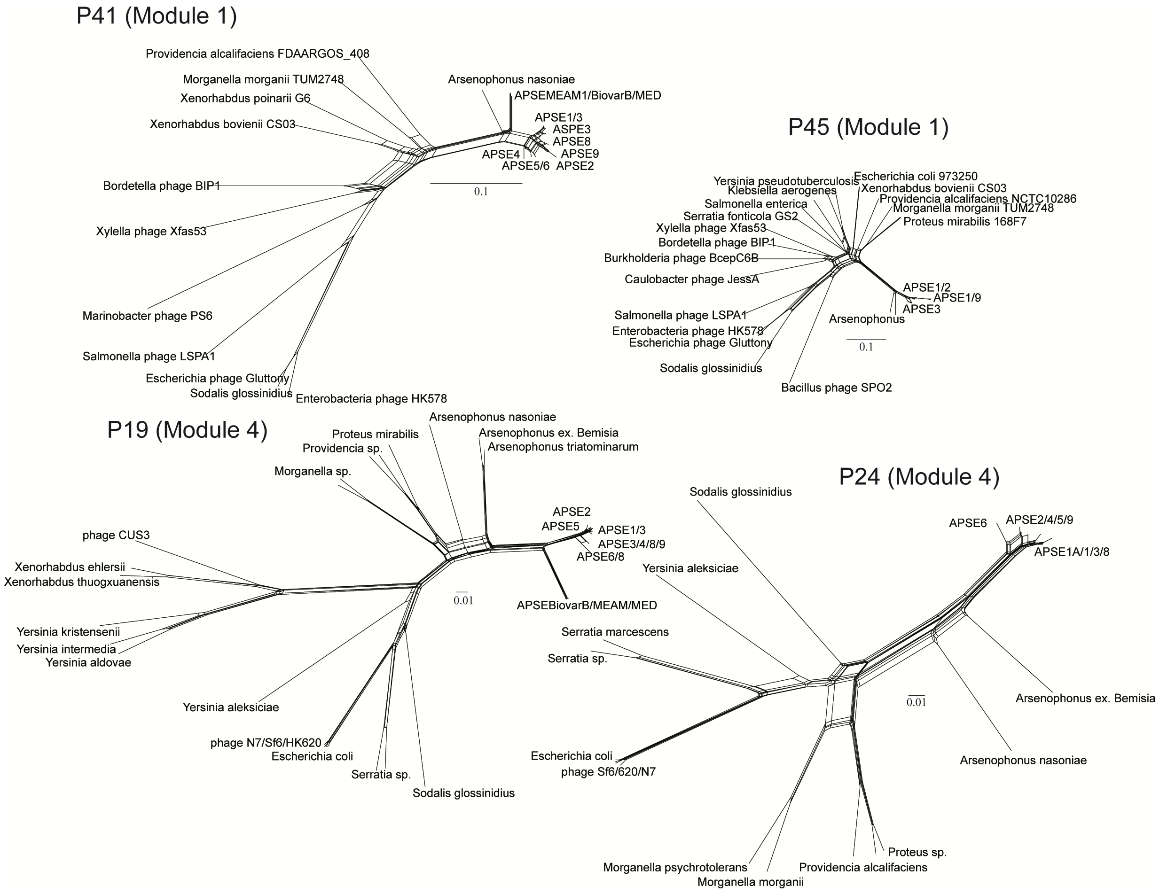

Figure S2. Phylogenetic networks depicting evolutionary relationships of the same genes analyzed in Fig. 5. Scale bars indicates nucleotide substitutions per site.

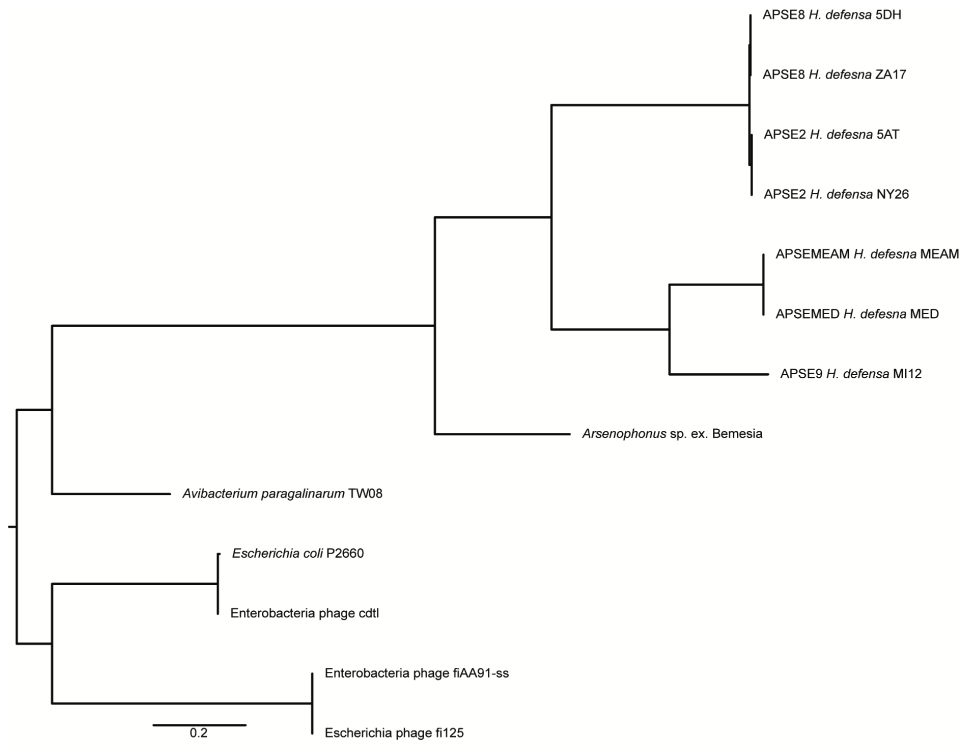

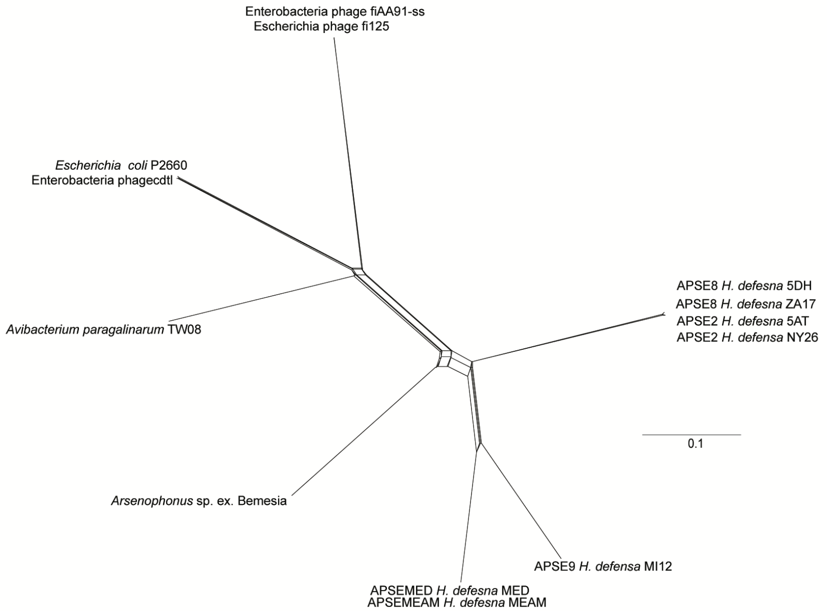

Fig. S3. Maximum-likelihood phylogram (A) and phylogenetic network (B) depicting evolutionary relationships for *ctdB* genes in different APSE haplotypes, other phages or phage elements in other bacteria in the order *Enterobacterales*.
